# Impact of Post-Cooking Storage on the Glycemic Profile of Boiled Rice: Integrating Glycemic Index, Resistant Starch, and Post-Technological Stability

**DOI:** 10.3390/foods15091472

**Published:** 2026-04-23

**Authors:** Rodica Siminiuc, Anna Vîrlan

**Affiliations:** Faculty of Food Technology, Technical University of Moldova, 168, Stefan cel Mare Bd., MD-2004 Chisinau, Moldova; anna.virlan@doctorat.utm.md

**Keywords:** postprandial response, starch retrogradation, carbohydrate digestibility, cold storage, freezing, incremental area under the curve

## Abstract

Post-cooking storage may modify the glycemic response of starchy foods; however, this effect is usually assessed only through the glycemic index (GI), without capturing the temporal dimension of the metabolic response. In this study, the effect of post-cooking storage on boiled rice was investigated using an integrated approach based on GI, resistant starch (RS) content, and the post-technological stability coefficient (PTSC). Storage significantly reduced GI, from 83.03 ± 15.02 (SD) in the freshly prepared sample to 43.55 ± 6.99 (SD) after prolonged freezing, while concurrently increasing RS from approximately 1.8% to nearly 4.0%. A strong inverse linear relationship was identified between RS and GI (r = −0.935, *p* < 0.001; R^2^ = 0.8735). These changes are consistent with storage-induced starch retrogradation and reduced enzymatic accessibility of the starch matrix. PTSC analysis further suggested that GI reduction was not automatically equivalent to lower temporal variability in the glycemic response: refrigeration was associated with more negative and more dispersed PTSC values, whereas prolonged freezing was associated with lower GI, higher RS, and smaller temporal variations in the incremental area under the curve (iAUC). Overall, the results suggest that the isolated analysis of GI may not fully describe the effect of post-cooking storage on boiled rice. The combined interpretation of GI, RS, and PTSC may provide a more informative framework for evaluating the metabolic effect of storage and may help differentiate between regimes predominantly associated with a reduction in the amplitude of the glycemic response and those additionally characterized by lower temporal variability of that response.

## 1. Introduction

The postprandial glycemic response to carbohydrate-rich foods is an important parameter in evaluating the metabolic effects of the diet, with implications for glycemic control, metabolic risk assessment, and the development of dietary recommendations [1]. In this context, the glycemic index (GI) is widely used to express the magnitude of the glycemic response elicited by a food. However, interpreting GI in isolation provides only a partial picture, as it does not directly capture either structural changes in the starch matrix or the temporal dynamics of the metabolic response [2].

Rice represents a relevant food model for this issue due to its widespread global consumption and the predominance of starch as the main carbohydrate constituent of the grain, with direct implications for digestibility and the glycemic response [3,4]. After cooking, the starch matrix of rice continues to evolve during storage, particularly under refrigeration and freezing conditions, through retrogradation and structural reorganization processes that modify the microstructural properties and digestive accessibility of starch [5,6]. These changes are generally attributed to the progressive reassociation of gelatinized starch chains, especially amylose, through hydrogen bonding, partial recrystallization, and the formation of more ordered domains that are less accessible to amylolytic enzymes [7]. One of the important consequences of these transformations is an increase in the resistant starch (RS) fraction, a component frequently associated with reduced starch digestibility and attenuation of the postprandial glycemic response [8,9].

In the scientific literature, the effect of post-cooking storage on starchy foods has been examined mainly through two types of outcomes: reduction in glycemic index or postprandial glycemic response and increase in resistant starch (RS) content [10,11,12,13]. Studies on rice and other starch-rich foods generally show that cooling, refrigeration, or freezing after cooking may attenuate glycemic response in parallel with starch retrogradation and increased formation of digestion-resistant fractions [12,14]. However, such an approach still captures the metabolic effect of post-technological treatments only partially. A lower GI value indicates attenuation of response amplitude, but it does not by itself clarify whether the postprandial response becomes more temporally stable or whether the observed reduction reflects a still-evolving restructuring process. This distinction is relevant because glycemic responses may show substantial intra-individual variability even under controlled repeated-meal conditions, as demonstrated in healthy adults without diabetes [15]. Given that post-cooking storage is common in household consumption, meal preparation in advance, and food systems based on refrigeration and freezing, the metabolic evaluation of starchy products should not be limited to a GI value determined at a single time point [16,17]. An integrated approach is therefore needed, capable of simultaneously capturing the amplitude of the glycemic response, its structural basis, and its temporal stability.

To address this gap, the present study proposes an integrated evaluation of the effect of post-cooking storage on boiled rice based on three complementary dimensions: GI, as an expression of the amplitude of the glycemic response; RS content, as a parameter associated with the structural reorganization of starch; and the post-technological stability coefficient (PTSC), which, in the context of this study, adds a descriptive dimension regarding the temporal variation in the response. Within this framework, GI expresses the intensity of the glycemic response, RS reflects the degree of restructuring of the starchy fraction, and PTSC complements the evaluation by characterizing the stability of the response after storage.

On this basis, the working hypothesis was that the storage regime influences not only the amplitude but also the temporal stability of the glycemic response of boiled rice. The integration of GI, RS, and PTSC was explored as a potentially more comprehensive approach to assessing the metabolic effect of storage than the isolated use of the glycemic index. In this context, the aim of the study was to evaluate how post-cooking storage conditions modify the glycemic response of boiled rice from the perspective of three complementary dimensions:

response amplitude, expressed by the glycemic index;
the structural component, reflected by resistant starch content;
temporal stability, assessed by the post-technological stability coefficient.

Specifically, the study aimed to evaluate the effect of different storage conditions on the glycemic index of boiled rice, to analyze the relationship between resistant starch content and variation in the glycemic index, and to characterize the temporal stability of the glycemic response through PTSC in order to differentiate storage conditions according to the observed glycemic profile.

## 2. Materials and Methods

The study was designed as a controlled, randomized, crossover experiment to evaluate the effect of post-cooking storage on the glycemic response of round-grain white rice (RGWR). For this matrix, postprandial glycemic response, GI, resistant starch (RS) content, and the post-technological stability coefficient were evaluated.

### 2.1. Ethical Considerations and Participants

The experimental protocol was approved by the Research Ethics Committee for Biomedical Studies of the Technical University of Moldova (protocol code 974, 15 November 2023). The study was conducted within the university’s Laboratory of Nutrition and Food Quality, and capillary blood glucose measurements were performed in collaboration with the Department of Gastroenterology of the Timofei Moșneaga Republican Clinical Hospital, Chișinău. The study was carried out in accordance with the ethical principles of the Declaration of Helsinki (2013 revision). Participation was voluntary, and all participants provided written informed consent before the first experimental session. Individual data were anonymized using numerical codes and stored in a secure electronic format accessible only to the research team.

The experimental study included 10 healthy adults (4 men and 6 women), aged between 20 and 35 years (25 ± 3 years), with body mass index within the normal range of 18.5–24.9 kg·m^−2^ (23.0 ± 1.6 kg·m^−2^), in accordance with the WHO guideline [18]. All participants were native residents of Chișinău, the capital of the Republic of Moldova, ensuring a homogeneous group with shared environmental and dietary influences. The inclusion criteria were the absence of diagnosed metabolic or gastrointestinal disorders, no use of medications or supplements affecting carbohydrate metabolism within the previous 14 days, non-smoking status, and willingness to comply with the experimental protocol. The exclusion criteria included type 1 or type 2 diabetes mellitus, celiac disease, major endocrine disorders, pregnancy or lactation, and non-compliance with fasting requirements before the experimental sessions [19].

### 2.2. Preparation of the Experimental Sample and Post-Cooking Storage Conditions

The RGWR variety was characterized by an amylose content of approximately 13% and amylopectin content of approximately 87%, based on the manufacturer’s technical documentation, with an available carbohydrate content of 77.8 g per 100 g dry weight. The initial resistant starch content of the freshly prepared sample, determined experimentally, was 1.78% (wet basis), as reported in Section 3.3. The RGWR variety was a commercially available milled white rice with no parboiling or other pre-treatment applied prior to use. The experimental sample was prepared from 64.3 g of rice, corresponding to a portion providing 50 g of available carbohydrates, cooked in 394 mL of water, equivalent to a rice-to-water ratio of 1:5.7, corresponding to the standardized preparation of liquid-consistency rice porridge [20]. The boiling time was 10 min, in accordance with the manufacturer’s instructions, timed from the moment continuous boiling of the water (100 °C) was reached. After cooking, the final mass of the sample was 463.0 g [19]. To investigate the influence of post-cooking storage on glycemic response and RS content, the rice sample was subjected to nine experimental conditions: the freshly prepared sample after standard cooling (S0), two storage conditions at room temperature (S6 and S12, at 20 °C), three refrigeration conditions (R24, R72, and R120, at 4 °C), and three freezing conditions (C720, C1440, and C2160, at −18 °C) (Table 1).

For samples stored at room temperature, the rice was kept on a tray at 20 °C for 6 or 12 h. Refrigerated samples were stored in airtight polypropylene containers at 4 °C for 24, 72, and 120 h. Frozen samples were stored hermetically at −18 °C for 720, 1440, and 2160 h, corresponding to 30, 60, and 90 days, respectively. Storage temperatures were monitored continuously throughout the experimental period using calibrated electronic data loggers. All storage regimes were designed to allow the progressive development of post-gelatinization transformations associated with starch retrogradation. Prior to consumption, all samples, regardless of storage condition, were reheated to 65–75 °C using a steam bath to ensure equivalent serving conditions across experimental sessions. This procedure was applied uniformly to the freshly prepared sample (S0) and to all stored samples (S6, S12, R24, R72, R120, C720, C1440, C2160). For each of the nine conditions, postprandial glycemic responses were measured, on the basis of which the incremental area under the curve and GI were calculated, while RS content was determined separately using an enzymatic method.

### 2.3. Glycemic Testing Protocol and Determination of Glycemic Index

Determination of the postprandial glycemic response was carried out under standardized conditions using a crossover experimental protocol. The operational parameters of the glycemic test are presented in Table 2. The protocol was applied in accordance with the principles of ISO 26642:2010 for the determination of the glycemic index of foods [21]. A solution containing 50 g of glucose dissolved in 250 mL of water was used as the reference food and administered according to the same experimental protocol.

After an overnight fast of 10–12 h, participants consumed either the test sample providing 50 g of available carbohydrates [22] or the glucose solution used as the reference. Capillary blood glucose was measured at 0, 15, 30, 45, 60, 90, and 120 min after sample ingestion. Capillary blood samples were collected from the fingertip using glass capillary tubes with a volume of approximately 40 µL and processed according to the laboratory protocol for glucose concentration determination. In cases where a measured value appeared aberrant, the determination was repeated immediately; if the repeated value was concordant with the first measurement, the mean of the two was used in calculations. No missing glucose values occurred across the experimental sessions, and all participants contributed complete glycemic response profiles for each condition. Glucose concentration was determined by the enzymatic glucose oxidase method (endpoint colorimetric method) using a Stat Fax^®^ 1904 semi-automated biochemical analyzer (Awareness Technology, Inc., Palm City, FL, USA). The method is based on the oxidation of glucose by glucose oxidase, with the formation of hydrogen peroxide, followed by a chromogenic reaction catalyzed by peroxidase and photometric reading of color intensity. Measurements were performed at a wavelength of 340 nm and at a temperature of 37 °C, in accordance with the manufacturer’s protocol [19].

For each experimental condition, the glycemic response was expressed as the incremental area under the curve (iAUC_0–120_), calculated using the trapezoidal rule based on blood glucose values measured at 0, 15, 30, 45, 60, 90, and 120 min after sample ingestion. Only the areas above the baseline value were included in the calculation, in accordance with the recommendations of ISO 26642:2010. The iAUC was determined according to the following equation:

(1) $$iAUC_{0–120}=\sum_{i=1}^{n-1}\;\frac{\left(G_{i}+G_{i+1}\right)}{2}\left(t_{i+1}-t_{i}\right)$$

where

$G_{i}$ represents the capillary blood glucose at time $t_{i}$;

n represents the total number of measurement points;

$G_{i+1}$ represents capillary blood glucose at time $t_{i+1}$; 

$t_{i}$ and $t_{i+1}$ represent two consecutive measurement time points (min).

The GI of each sample was calculated as the ratio between the incremental area obtained for the test sample and the incremental area obtained for the reference glucose solution, according to the following equation:

(2) $$GI=\frac{iAUC_{sample}}{iAUC_{glucose}}\times 100$$

The glycemic index value of the glucose solution was set at 100, and the GI values of the experimental samples were expressed relative to this reference.

### 2.4. Determination of Resistant Starch Content

The RS content of the samples was determined by an enzymatic method based on the procedure described by Englyst, Kingman, and Cummings [23], used for the quantification of nutritionally relevant starch fractions. For the analysis, 0.5 g of wet sample was used and incubated with 4.0 mL of an enzymatic solution containing pancreatic α-amylase (10 mg/mL) and amyloglucosidase (AMG, 3 U/mL; enzyme kit E-AMGDF, Megazyme International Ireland, Bray, Ireland) for 16 h at 37 °C in a thermostated shaking water bath (OLS200, Grant Instruments, Cambridge, UK; 200 rpm) to hydrolyze the digestible starch fraction.

The enzymatic reaction was stopped by adding 4.0 mL of 99% ethanol, and the RS fraction was recovered by centrifugation at approximately 3000 rpm for 10 min at room temperature. The precipitate was washed twice with 50% ethanol, followed by decantation, and excess liquid was removed with filter paper. The resistant fraction was then solubilized in 2 mL of 2 M KOH under vigorous magnetic stirring in an ice bath for 20 min, neutralized with 8 mL of 1.2 M sodium acetate buffer (pH 3.8), and subjected to final hydrolysis with 0.1 mL AMG (3300 U/mL) for 30 min at 50 °C in a thermostated shaking water bath (200 rpm).

The concentration of released glucose was determined by the glucose oxidase–peroxidase (GOPOD) method using a Stat Fax^®^ 1904 semi-automated biochemical analyzer (Awareness Technology, Inc., Palm City, FL, USA). RS content was expressed as a percentage of the analyzed sample mass.

### 2.5. Calculation of the Post-Technological Stability Coefficient (PTSC)

To evaluate the temporal variation in the glycemic response during storage of the prepared product, the post-technological stability coefficient (PTSC) was calculated. In the context of this study, this indicator was used to express the relative daily variation in the incremental area of the glycemic response in relation to the freshly prepared sample and to compare changes in the metabolic response across the experimental storage regimes. The PTSC was calculated according to the following equation:

(3) $$PTSC=\frac{iAUC_{t}-iAUC_{0}}{iAUC_{0}}\times \frac{100}{t}$$

where

$iAUC_{0}$ represents the incremental area of the glycemic response for the freshly prepared sample;

$iAUC_{t}$ represents the incremental area of the glycemic response after a storage time of t;

t represents storage duration, expressed in days.

During data processing, storage durations initially expressed in hours were converted into days for PTSC calculation. PTSC values were expressed as the percentage change in iAUC per day (%·day^−1^). Values close to zero indicate limited temporal variation in the glycemic response relative to the freshly prepared sample, whereas more negative values indicate a more pronounced reduction in iAUC during storage. In the context of this study, PTSC was used together with GI and RS content for the integrated evaluation of response amplitude, structural support, and temporal variation in the glycemic response.

It should be noted that PTSC, as applied in this study, functions as a descriptive indicator of the rate of change in glycemic response relative to the freshly prepared sample, rather than as an externally validated measure of stability. The rationale for its use is grounded in the iAUC framework established by ISO 26642:2010 [21]: by normalizing the difference in iAUC to storage duration, PTSC captures the daily rate of glycemic change in a comparable, dimensionally consistent manner. As a consequence of its formulation, PTSC values are sensitive to the storage interval used as a reference point, meaning that comparisons across studies with markedly different storage durations should be made with caution. External validation against independent measures of glycemic stability represents a direction for future work. Negative PTSC values indicate a reduction in iAUC relative to the freshly prepared sample but do not necessarily imply that structured starch retrogradation has occurred; the observed reduction may also reflect inherent intra-individual variability in glycemic response between measurement sessions.

### 2.6. Statistical Analysis

Experimental data were analyzed using descriptive and inferential statistical methods. Results were expressed as mean ± standard deviation to characterize the central tendency and variability of the obtained values. Individual glycemic index values were compared across storage conditions using one-way repeated-measures ANOVA, with each participant assessed under all experimental conditions. When the assumption of sphericity was violated, the Geisser–Greenhouse correction was applied. Multiple comparisons against the control condition (S0, freshly prepared) were performed using Dunnett’s post hoc test. Statistical analysis of GI data was conducted in GraphPad Prism 11.0.0 (GraphPad Software, San Diego, CA, USA).

For PTSC, differences between storage times were analyzed separately within the refrigeration and freezing regimes using the Friedman test for repeated measures, followed, when appropriate, by Dunn’s post hoc test for multiple comparisons. RS content was determined in triplicate for each storage condition, and the overall effect of storage conditions was evaluated by one-way ANOVA. The relationship between RS content and GI was assessed using Pearson’s correlation coefficient and simple linear regression. The goodness of fit of the regression model was evaluated by the coefficient of determination (R^2^). Microsoft Excel for Mac (version 16.107.2, Microsoft Corporation, Redmond, WA, USA) was used for data organization, descriptive analyses, and preliminary calculations. The threshold for statistical significance was set at *p* < 0.05.

## 3. Results

### 3.1. Effect of Storage Conditions on the Glycemic Index of Boiled Rice

To evaluate the effect of post-cooking storage on the glycemic response, variation in the glycemic index of boiled rice was analyzed as a function of storage temperature and duration. The obtained values showed a progressive reduction in GI, with different amplitudes across the investigated regimes.

**Figure 1:**
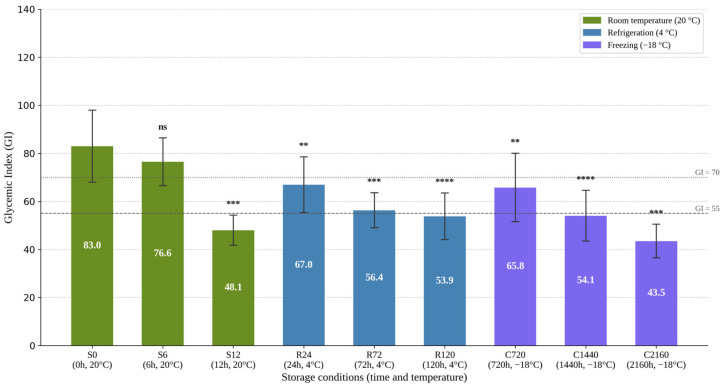
Mean glycemic index (GI ± SD) as a function of the post-cooking storage regime of the RGWR sample. Legend: Error bars indicate standard deviation (SD, *n* = 10). Significance vs. S0 (Dunnett’s post hoc test): ns: *p* > 0.05; **: *p* ≤ 0.01; ***: *p* ≤ 0.001; ****: *p* ≤ 0.0001. RGWR = round-grain white rice.

**Variation in mean GI values across regimes**. GI ranged from 83.03 ± 15.02 (SD) for the freshly prepared sample to 43.55 ± 6.99 after prolonged storage at −18 °C, corresponding to an overall reduction of approximately 48%. The distribution of mean values indicates that post-cooking history substantially influences the glycemic response of the product (Figure 1).

The trajectory of GI reduction depended on the applied storage regime. At 20 °C, the decrease was rapid, with the GI value reaching 48.08 ± 6.28 after 12 h, suggesting the early onset of changes in the starch fraction after cooking. Under refrigeration conditions (4 °C), the decrease followed a gradual profile, from 66.98 ± 11.62 after 24 h to 53.86 ± 9.73 after 120 h. In the case of freezing (−18 °C), marked reductions in GI became evident mainly after extended storage, with the minimum value reached at 2160 h. Overall, these differences show that the effect of storage on GI is conditioned by both temperature and duration of exposure. In relative terms, storage at 20 °C for 12 h resulted in a GI reduction of approximately 42% compared with the freshly prepared sample, whereas refrigeration for 120 h produced a reduction of approximately 35%.

**Confirmation of the overall effect of the storage regime on GI.** To verify whether the observed variations exceeded the inherent variability of in vivo testing, individual glycemic index values were analyzed by one-way repeated-measures ANOVA with Geisser–Greenhouse correction. The analysis confirmed a significant overall effect of storage conditions on glycemic index, F(2.795,25.16) = 26.46, *p* < 0.0001. The effect of interindividual variability among participants was also significant, F(9,72) = 7.526, *p* < 0.0001, indicating the presence of relevant interindividual variability, which was nevertheless controlled for by the analytical design.

To identify the conditions associated with a significant reduction in glycemic index relative to the control sample, Dunnett’s post hoc test was applied using the freshly prepared sample (0 h, 20 °C) as the reference. The analysis showed that storage for 6 h at 20 °C did not significantly modify GI relative to the control (*p* = 0.1730). In contrast, all other conditions produced significant reductions in GI compared with the control: after 12 h at 20 °C (*p* = 0.0008), after 24 h, 72 h, and 120 h at 4 °C (*p* = 0.0011, *p* = 0.0004, and *p* < 0.0001, respectively), as well as after 720 h, 1440 h, and 2160 h at −18 °C (*p* = 0.0080, *p* < 0.0001, and *p* = 0.0004, respectively). Therefore, the reduction in glycemic index became significant after 12 h at 20 °C, after 24 h under refrigeration, and for all investigated durations under freezing conditions, with the most pronounced effect observed after 2160 h at −18 °C.

### 3.2. Evaluation of the Temporal Stability of the Glycemic Response Through the PTSC

**Figure 2:**
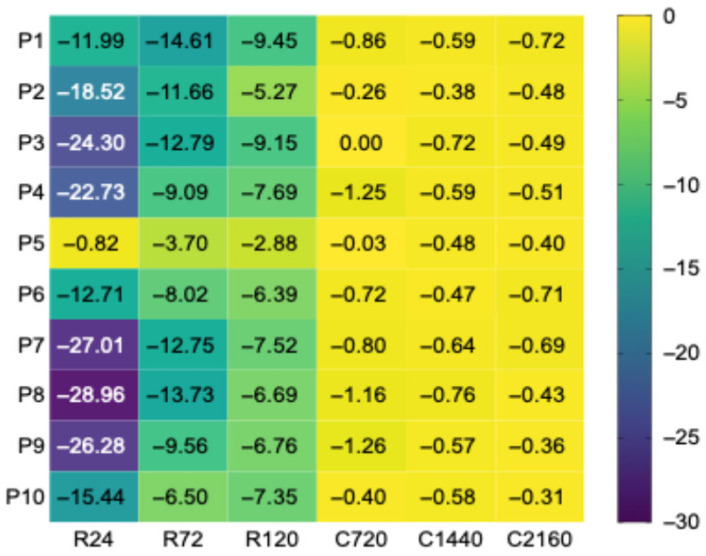
Heatmap of individual PTSC values (ΔiAUC, %/day) for samples stored under refrigeration and freezing conditions. Legend: Each cell represents the value of the post-technological stability coefficient (PTSC), expressed as the relative daily variation in the incremental area under the curve (ΔiAUC, %/day), for one participant (P1–P10). On the horizontal axis, R indicates refrigeration regimes at 4 °C (R24, R72, R120), whereas C indicates freezing regimes at −18 °C (C720, C1440, C2160); the numerical values represent storage duration, expressed in hours. The color scale reflects the amplitude of the daily variation in iAUC: values close to 0 indicate limited temporal variation, whereas more negative values indicate more pronounced reductions in iAUC during storage.

To extend the evaluation based on GI, the temporal variation in the glycemic response was analyzed using the PTSC, expressed as the relative daily variation in the incremental area under the curve (ΔiAUC, %/day). The data reveal a clear contrast between the two types of storage. Under refrigeration conditions (4 °C), PTSC values showed greater amplitudes and marked interindividual variability, ranging from −0.82 to −28.96%/day, suggesting that the glycemic response continued to change between successive storage intervals. In contrast, under freezing conditions (−18 °C), PTSC values were lower in amplitude and much more homogeneous across participants, with most values falling approximately between −0.31 and −0.86%/day, suggesting a much lower daily rate of change in iAUC (Figure 2).

To assess whether the descriptive differences corresponded to significant variations across storage times, PTSC values were analyzed separately within each thermal regime using the Friedman test for repeated measures, followed by Dunn’s post hoc comparisons (Table 3).

Under the refrigeration regime, the analysis revealed significant differences between storage times (χ^2^ = 11.40; *p* = 0.0020), with the significant difference observed between R24 and R120 (*p* = 0.0024), whereas the comparisons R24 vs. R72 and R72 vs. R120 did not reach statistical significance. Under the freezing regime, the Friedman test did not indicate significant differences between the analyzed time points (χ^2^ = 1.80; *p* = 0.4362), and post hoc comparisons revealed no significant differences between any of the investigated durations. These results suggest greater temporal variability in PTSC values during refrigeration and relatively greater stability during freezing.

A direct comparison confirmed a significant difference between the two thermal regimes (Wilcoxon signed-rank test: W = 0, *p* = 0.002; median PTSC: −0.67%/day for freezing vs. −12.59%/day for refrigeration), with freezing consistently associated with smaller daily variations in iAUC across all 10 participants (Supplementary Tables S1 and S2).

### 3.3. Evolution of Resistant Starch Content as a Function of the Storage Regime

**Figure 3:**
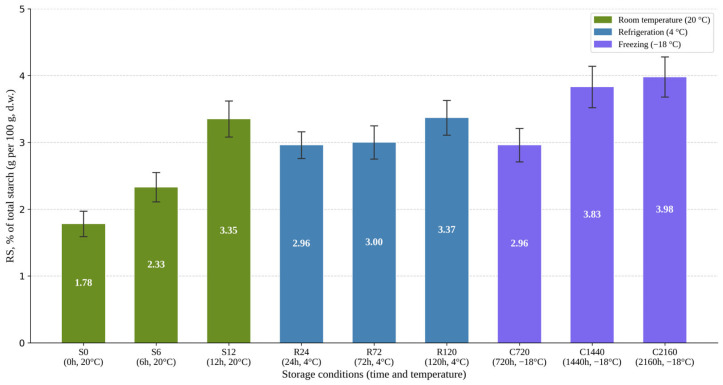
Resistant starch content (%) of the RGWR sample as a function of the post-cooking storage regime. Legend: RS values are expressed as mean ± SD, calculated on the basis of three independent determinations (*n* = 3) for each storage condition; RGWR = round-grain white rice; RS = resistant starch.

To evaluate the structural component associated with the metabolic changes observed previously, the evolution of RS content was analyzed as a function of the post-cooking storage regime. RS content increased during storage of the product, although with distinct trajectories depending on storage temperature and duration. The freshly prepared sample showed the lowest RS level, approximately 1.78%. At 20 °C, RS content increased rapidly during the early post-cooking interval, reaching approximately 3.35% after 12 h (Figure 3).

This rapid increase is consistent with the preferential retrogradation of amylose chains, which, following gelatinization, reassociate rapidly through hydrogen bonding and recrystallization even at ambient temperature, forming digestion-resistant structures classified as RS3. Amylopectin retrogradation, by contrast, proceeds more slowly and is generally associated with longer storage times and lower temperatures.

Under refrigeration conditions (4 °C), RS values remained within a relatively narrow range of approximately 2.96–3.37% throughout the investigated regimes. In the case of freezing (−18 °C), the highest RS values were recorded, with prolonged storage leading to levels close to 4%, reaching a maximum of 3.98% after 2160 h. Overall, the results indicate a rapid initial increase in RS after cooking, followed by the maintenance of elevated levels under cold storage regimes, with the highest values observed under prolonged freezing conditions.

**Overall effect of storage conditions on resistant starch content.** To verify whether the observed variations in RS content reflected systematic effects of the storage regime rather than experimental variability between determinations, RS% values were analyzed by one-way ANOVA. The results revealed a significant overall effect of storage conditions on RS content, F(8,18) = 23.25, *p* = 5.56 × 10^−8^, confirming that the storage regime significantly influences the RS content of the RGWR sample.

### 3.4. Relationship Between Resistant Starch Content and Glycemic Response

**Figure 4:**
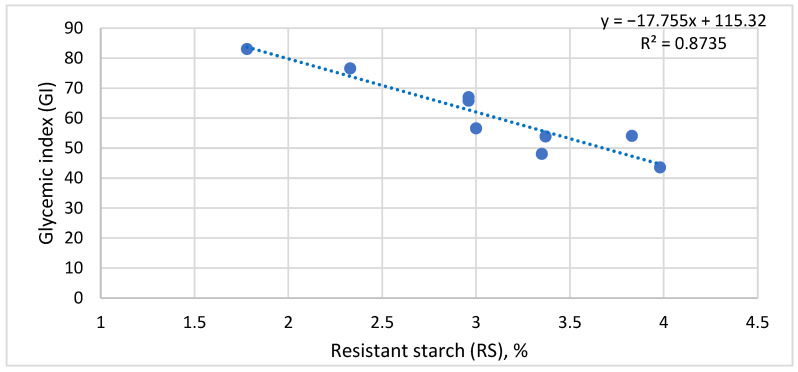
Relationship between mean glycemic index (GI) and resistant starch content (RS%) as a function of the storage regime. Legend: Each point represents the mean GI–RS% pair corresponding to one storage condition (*n* = 9). The dotted line indicates the linear regression (r = −0.935, R^2^ = 0.8735, *p* < 0.001). Samples characterized by RS values below 2.5% were associated with high glycemic index values, whereas the range of approximately 3–4% RS corresponded to moderate or reduced GI values. This pattern indicates that the accumulation of resistant starch is associated with attenuation of the glycemic response of the product after storage. The results support the existence of a close functional link between the structural reorganization of starch and the glycemic behavior of boiled rice, thereby justifying the integrated analysis of metabolic and structural parameters in the following section.

To correlate the structural and metabolic results obtained previously, the relationship between mean GI and the corresponding RS content across the nine investigated storage regimes was analyzed. Pearson’s correlation coefficient was r = −0.935 (*p* < 0.001), and the coefficient of determination was R^2^ = 0.8735, indicating that a large proportion of the variation in glycemic response was associated with changes in RS content (Figure 4).

## 4. Discussion

The results of this study show that post-cooking storage modifies not only the amplitude of the glycemic response but also its temporal stability and that this distinction becomes more intelligible through the integration of metabolic and structural parameters.

### 4.1. Effect of Post-Cooking Storage on the Amplitude and Stability of the Glycemic Response

Post-cooking storage significantly modifies the glycemic response of boiled rice, although the magnitude and consistency of this effect differ across the investigated regimes. The reduction in glycemic index observed after storage is consistent with previous studies showing that cooling and cold storage of cooked rice may attenuate postprandial glycemic response, particularly when post-cooking treatment promotes starch retrogradation and an increase in digestion-resistant fractions. In this regard, lower postprandial glycemic responses after consumption of cooled or reheated rice have been reported together with evidence of increased resistant starch formation after refrigeration [14,24,25].

However, the present data also indicate that a reduction in the amplitude of the glycemic response is not, by itself, sufficient to fully characterize the metabolic behavior of the product after storage. In the analyzed experimental set, some storage regimes led to lower GI values without being accompanied by comparable stabilization of the response over time, whereas others led to lower GI values with reduced temporal variation in iAUC. From this perspective, the amplitude of the glycemic response and its temporal stability should be interpreted as complementary rather than equivalent dimensions. A lower GI reflects attenuation of response magnitude, but it does not by itself show whether the postprandial profile has become more stable or whether the observed reduction reflects a still-evolving restructuring process [7].

This distinction is supported by recent evidence showing that postprandial glycemic responses to duplicate meals may exhibit substantial intra-individual variability even under controlled conditions [15]. In this context, PTSC adds a useful descriptive dimension by helping to distinguish between storage regimes associated mainly with a lower response amplitude and those additionally associated with reduced temporal variability. The present results therefore extend previous observations on storage-treated rice by suggesting that post-cooking history influences not only the magnitude of the glycemic response but also its temporal behavior, and that interpretation based on GI alone may remain incomplete when post-technological restructuring is still evolving [12,26].

### 4.2. Resistant Starch as the Structural Basis of the Observed Metabolic Changes

**Figure 5:**
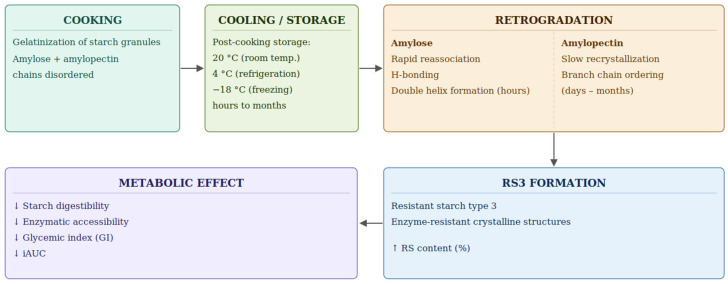
Schematic representation of post-cooking starch retrogradation and resistant starch formation. Legend: RS3 = resistant starch type 3; GI = glycemic index; iAUC = incremental area under the curve. Source: own elaboration.

The increase in RS content observed after post-cooking storage provides a plausible structural basis for the metabolic changes identified in this study. In cooked starchy systems, cooling and storage promote retrogradation after gelatinization, with progressive reassociation and recrystallization of starch chains, especially amylose, leading to the formation of digestion-resistant fractions, particularly RS3 (Figure 5).

Structurally, the retrogradation of gelatinized starch proceeds through two kinetically distinct processes. Amylose, owing to its predominantly linear conformation, reassociates rapidly within the first hours after cooling through the formation of double-helical junction zones, constituting the principal molecular basis of RS3 [7,27]. Amylopectin retrogradation, by contrast, involves the slower reordering of its shorter outer branches through both intra- and intermolecular interactions, typically requiring days to weeks of cold storage to produce measurable increases in crystallinity [27,28]. In rice-based systems, this two-phase retrogradation pattern may be further modulated by non-starch constituents of the grain matrix, particularly endogenous proteins. Recent studies have shown that rice protein can retard starch retrogradation by competing for available water within the gel matrix, restricting the mobility of starch chains through hydrogen bonding and steric interactions, and reducing the rate of recrystallization during cold storage [29,30]. These protein–starch interactions suggest that retrogradation kinetics and RS3 formation in whole-grain rice systems may differ from those observed in purified starch models and that the extent of structural reorganization during storage reflects the combined influence of starch molecular architecture and the surrounding grain matrix composition [30,31].

These structural changes reduce enzymatic accessibility and slow starch hydrolysis, thereby providing a biologically plausible explanation for why cooled or stored starchy foods may elicit a lower glycemic response than freshly prepared products [32,33,34].

In the present study, the evolution of RS followed the same general direction as the observed metabolic changes: regimes associated with higher resistant starch levels generally corresponded to lower glycemic index values. This interpretation is consistent with previous studies on rice, showing that storage conditions associated with higher RS formation may also be accompanied by lower glycemic response or glycemic load compared with freshly prepared products [12,24,25]. The inverse linear relationship between RS and GI identified in Section 3.4 therefore supports the interpretation that structural reorganization of starch represents one of the major components underlying the reduction in glycemic response after storage.

The present results also suggest that RS should not be interpreted exclusively as a marker of reduced glycemic response amplitude but also as an indicator of the structural transformations associated with storage. From this perspective, RS accumulation may be interpreted as an expression of the progressive reorganization of the starch matrix, capable of modifying both digestive accessibility and the glycemic response of the product during storage. Thus, resistant starch may be regarded as a linking element between post-cooking structural modification and the metabolic response observed experimentally.

### 4.3. Refrigeration Versus Freezing: Transient Reduction Versus More Advanced Stabilization

The direct comparison of the two storage regimes suggests that the reduction in glycemic response does not have the same functional significance under refrigeration and freezing. In our data, refrigeration was associated with a decrease in glycemic index and an increase in resistant starch, but it maintained more negative PTSC values and greater temporal variation, suggesting that the glycemic response remained less stable over time under these conditions. In contrast, prolonged freezing was associated simultaneously with lower GI values, higher RS levels, and smaller temporal variations in iAUC, corresponding to a more homogeneous glycemic response profile over time. These differences indicate that a reduction in the amplitude of the glycemic response and a reduction in its temporal variability are not completely overlapping processes but may follow different trajectories depending on post-cooking thermal history [26,28].

For refrigeration, the literature relatively consistently supports a reduction in glycemic response after cooling cooked rice [14,24,25]. Increases in resistant starch and reductions in glycemic response have been reported for cooled rice, including after reheating, which is in line with the general direction of our results. However, the present data additionally suggest that, under certain conditions, GI reduction during refrigeration may coexist with still appreciable temporal variability in the glycemic response [14,26].

For freezing, the literature provides mainly structural rather than directly metabolic explanations. Recent studies indicate that the retrogradation behavior of starch in rice-based systems depends on time, temperature, and thermal history and that the formation of RS3 results from gelatinization followed by retrogradation and recrystallization. In this context, the present results support the interpretation that refrigeration may be associated with a still-evolving reorganization of the starch system, whereas prolonged freezing may be compatible with a more advanced or more stable post-storage organization, reflected here by PTSC values closer to zero [28,35,36]. From this perspective, the present experimental set differentiates between a transient reduction in the glycemic response, more closely associated with refrigeration, and a lower temporal variability of that response, more compatible with prolonged freezing, in line with the view that post-cooking thermal history may shape distinct trajectories of starch reorganization and metabolic expression [26,28].

Within this regime, the most pronounced convergence of these parameters was observed for the sample stored for 2160 h (C2160), which showed the lowest mean GI (43.55 ± 6.99), the highest RS content (3.98%), and the most homogeneous PTSC values across all participants. Although the Friedman test did not reveal significant differences among the three freezing durations (*p* = 0.4362), the progressive decrease in GI and concurrent increase in RS from C720 to C2160 suggest a continued, gradual accumulation of digestion-resistant structures over the 90-day storage period. From a structural perspective, this pattern is consistent with the time-dependent progression of starch retrogradation at sub-zero temperatures: while amylose reassociation occurs predominantly during the early post-cooling period, the continued increase in RS observed between C720 and C2160 is compatible with the slower recrystallization of amylopectin outer branches, which has been shown to proceed over weeks to months under cold storage conditions [27,36]. At −18 °C, the severely restricted molecular mobility may limit the formation of new nucleation sites while still permitting the growth and perfection of existing crystalline domains [5,28], resulting in a starch matrix that becomes progressively more ordered and less accessible to enzymatic hydrolysis.

The RGWR variety used in this study, with an amylose content of approximately 13%, falls below the threshold of approximately 20% at which the lowest nucleation rate has been reported for rice starches during long-term retrogradation [27], suggesting that nucleation may proceed relatively faster in this starch system, with the prolonged storage period at −18 °C primarily contributing to crystal growth and perfection rather than to the initiation of new ordered domains. Taken together, these observations suggest that the glycemic profile of C2160 reflects a structurally more advanced stage of post-cooking starch reorganization, in which both the amplitude and temporal stability of the glycemic response converge toward lower and more homogeneous values.

It should be noted that all samples in this study were reheated to 65–75 °C prior to consumption. At this temperature range, moderate reheating may partially disrupt amylopectin crystallites formed during retrogradation, potentially attenuating some of the RS-related effects on starch digestibility and glycemic response [24], while the overall structural modifications accumulated during storage are largely preserved [5]. The consistent application of this procedure across all experimental conditions ensured comparability of the measured glycemic responses.

### 4.4. Integrative Conceptual Model of GI–RS–PTSC

**Figure 6:**
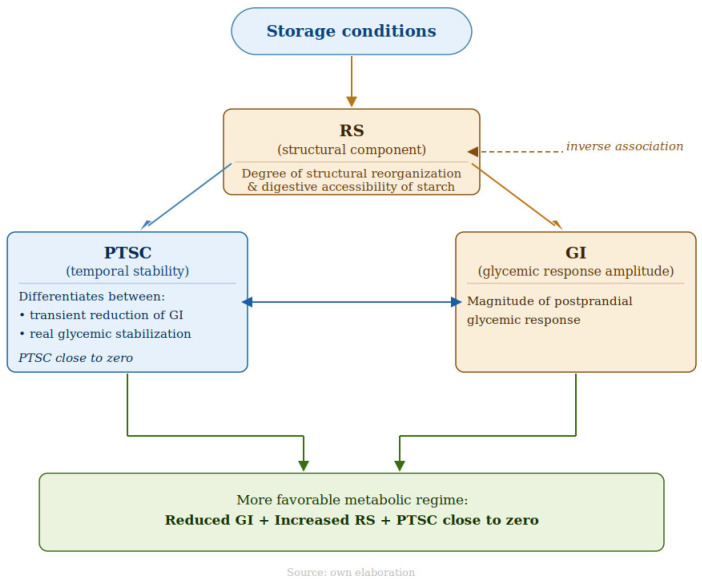
Integrative conceptual model of the relationship between glycemic index (GI), resistant starch (RS) content, and the post-technological stability coefficient (PTSC). Legend: The model synthesizes the relationships observed between storage conditions, structural changes indirectly reflected by RS, and the glycemic response of the product. An increase in RS content is associated with a reduction in the amplitude of the glycemic response, whereas PTSC values close to zero correspond to lower temporal variation in iAUC.

To synthesize the relationships observed among the analyzed parameters, the experimental results may be interpreted in relation to three complementary dimensions: the amplitude of the glycemic response, represented by GI; the structural component, indirectly reflected by RS content; and the temporal dimension of the response, captured by PTSC. A conceptual synthesis of these relationships is presented in Figure 6.

The present results show that a reduction in glycemic index is associated with an increase in RS content; however, this change does not appear sufficient, when considered in isolation, to describe the temporal dynamics of the glycemic response. Under refrigeration, the decrease in GI and increase in RS were accompanied by more negative and more dispersed PTSC values, whereas prolonged freezing showed lower GI, higher RS levels, and smaller temporal variations in iAUC, compatible with a more homogeneous glycemic response profile over time. Within this dataset, the integration of GI, RS, and PTSC made it possible to differentiate between regimes predominantly associated with a reduction in the amplitude of the glycemic response and those additionally characterized by lower temporal variability of that response, with prolonged freezing showing the lowest GI values, the highest RS levels, and the most homogeneous PTSC values.

### 4.5. Novelty, Relevance, and Limitations of the Study

The present study extends existing approaches to evaluating post-cooking storage by introducing PTSC as a descriptive complement to GI and RS content, enabling simultaneous characterization of response amplitude, structural reorganization, and temporal variation in the glycemic response. While GI and RS have been individually examined in the context of starchy food storage, their combined interpretation with a temporal stability indicator represents a less frequently adopted analytical framework [26,37].

The study nevertheless has clear limitations. The analysis was conducted on a single food matrix and a single product type, which limits direct generalization of the conclusions to other starchy sources. The menstrual cycle phase was not controlled for female participants, which may have introduced variability in glycemic responses, given the known influence of hormonal fluctuations on insulin sensitivity and glucose metabolism.

In addition, the sample size and experimental design support the mechanistic and integrative character of the study but do not yet allow the formulation of universal conclusions. The GI–RS–PTSC conceptual model proposed here should therefore be interpreted as an integrative synthesis derived from the present dataset rather than as an externally validated predictive model. Validation across other rice types, other starchy foods, and larger cohorts represents a necessary step for future studies [38].

A specific limitation concerns the PTSC indicator: as a descriptive metric derived from iAUC values at two time points relative to the freshly prepared sample, it has not been validated against independent measures of glycemic stability, and its values are sensitive to the choice of storage interval, which limits direct comparability across studies with different storage durations. Furthermore, the structural interpretation proposed in this study is based on RS content as an indirect marker of starch reorganization, without direct instrumental confirmation of crystalline structure or retrogradation enthalpy. While the strong inverse relationship between RS and GI supports the plausibility of this interpretation, direct structural characterization would strengthen the mechanistic conclusions.

These limitations point to several directions for future research. At the methodological level, characterization of amylopectin retrogradation kinetics by differential scanning calorimetry (DSC), complemented by X-ray diffraction (XRD) analysis of crystalline polymorphism, would provide a more detailed structural basis for interpreting the observed glycemic changes. Additionally, the role of endogenous rice proteins in modulating retrogradation kinetics and RS3 formation, discussed in Section 4.2 on the basis of recent literature, warrants direct experimental investigation in the context of whole-grain rice systems stored under the conditions examined here.

At the applied level, the present findings are relevant for two practical directions: the optimization of post-cooking conditions for freshly consumed rice, where short-term storage at controlled temperatures may modulate the glycemic response without compromising sensory quality; and the development of RS-enriched rice products, where prolonged cold storage represents a feasible processing strategy for increasing resistant starch content and attenuating the postprandial glycemic response.

## 5. Conclusions

The results of this study suggest that the effect of post-cooking storage on the glycemic response of boiled rice cannot be adequately interpreted through GI considered in isolation. Reduction in the amplitude of the glycemic response reflects only part of the metabolic behavior of the product, as its significance appears to depend on both structural transformations of starch and the temporal variation in the postprandial response.

The integrated analysis of GI, RS content, and PTSC indicated that the amplitude of the glycemic response and its temporal variability represent complementary rather than equivalent dimensions. Within this framework, RS may be interpreted as reflecting the structural component associated with the observed metabolic changes, whereas PTSC allowed differentiation between a transient reduction in the glycemic response and a lower temporal variability of that response within the present experimental conditions.

Under the experimental conditions analyzed, refrigeration was associated with a reduction in GI without a comparable reduction in temporal variability, whereas prolonged freezing was associated with the lowest GI values, the highest RS levels, and the most homogeneous PTSC values. Overall, the study supports the usefulness of an integrated structural–metabolic approach for interpreting the effect of post-cooking storage on the glycemic behavior of boiled rice and, more cautiously, as a conceptual basis for the study of other starchy foods.

## Figures and Tables

**Table 1:** Experimental post-cooking storage conditions applied to the RGWR sample.

Sample Code	Post-Cooking Treatment	Temperature	Duration	Determinations Performed
S0	freshly prepared sample after standard cooling	20 °C	0 h	GR, GI, RS
S6	tray storage	20 °C	6 h	GR, GI, RS
S12	tray storage	20 °C	12 h	GR, GI, RS
R24	refrigeration in an airtight PP container	4 °C	24 h	GR, GI, RS
R72	refrigeration in an airtight PP container	4 °C	72 h	GR, GI, RS
R120	refrigeration in an airtight PP container	4 °C	120 h	GR, GI, RS
C720	airtight freezing	−18 °C	720 h (30 days)	GR, GI, RS
C1440	airtight freezing	−18 °C	1440 h (60 days)	GR, GI, RS
C2160	airtight freezing	−18 °C	2160 h (90 days)	GR, GI, RS

Note: S = storage at room temperature; R = refrigeration; C = freezing; GI = glycemic index; RS = resistant starch; GR = glycemic response; PP = polypropylene; RGWR = round-grain white rice.

**Table 2:** Operational parameters of the glycemic test.

Operational Parameter	Fixed Value
Minimum interval between sessions	≥7 days
Testing schedule	07:30–10:30
Pre-test fasting period	10–12 h
Maximum time allowed for sample consumption	12 ± 2 min
Water served concurrently	250 mL (22 ± 1 °C)
Restrictions during the 24 h preceding the test	alcohol, caffeine, physical activity > 3 MET
Diet control on the previous day	unrestricted diet, without foods rich in RS

Note: MET = metabolic equivalent of activity; 1 MET corresponds approximately to energy expenditure at rest.

**Table 3:** Results of the Friedman test and Dunn’s post hoc comparisons for PTSC values.

Thermal Regime	Time Points Analyzed	*n*	Friedman χ^2^	Global *p*	Dunn’s Post Hoc Comparison	Difference in Rank Sums	Z	Adjusted *p*
Refrigeration (4 °C)	R24, R72, R120	10	11.40	0.0020	R24-R72	−6.00	1.342	0.5391
					R24-R120	−15.00	3.354	0.0024
					R72-R120	−9.00	2.012	0.1325
Freezing (−18 °C)	C720, C1440, C2160	10	1.80	0.4362	C720-C1440	−3.00	0.6708	>0.9999
					C720-C2160	−6.00	1.342	0.5391
					C1440-C2160	−3.00	0.6708	>0.9999

Note: R = refrigeration; C = freezing; PTSC = post-technological stability coefficient, expressed as the relative daily variation in the incremental area under the curve (ΔiAUC, %/day). The Friedman test was applied separately to evaluate differences between storage times within each thermal regime, and pairwise comparisons were performed using Dunn’s post hoc test. Differences were considered significant at *p* < 0.05.

## Data Availability

The original contributions presented in this study are included in the article/Supplementary Materials. Further inquiries can be directed to the corresponding author.

## Supplementary Materials

The following supporting information can be downloaded at: https://www.mdpi.com/article/10.3390/foods15091472/s1, Table S1: Raw PTSC values per participant for refrigeration and freezing conditions; Table S2: Wilcoxon signed-rank test results for the comparison of PTSC values between thermal regimes.
